# Nomogram combining spectral dual-layer detector CT radiomics and deep learning features predicts alveolar tumor spread

**DOI:** 10.3389/fonc.2026.1703790

**Published:** 2026-04-10

**Authors:** Fanger Li, Changxing Fang, Aiqi Qiao, Yongxia Hao, Xiaomei Yao, Siyu Yang, Zengyu Jiang, Sheng He, Ying Qiao

**Affiliations:** 1 Department of Radiology, First Hospital of Shanxi Medical University, Taiyuan, China; 2 College of Medical Imaging, Shanxi Medical University, Taiyuan, China; 3 Department of Radiology, Cancer Hospital of Shanxi Medical University, Taiyuan, China; 4 Department of Pathology, First Hospital of Shanxi Medical University, Taiyuan, China; 5 Ministry of Education (MOE) Key Laboratory of Coal Environmental Pathogenicity and Prevention, The Transformation Pilot-Base of Shanxi Clinical Cell Therapy, Collaborative Innovation Center for Frontier Medicine, Shanxi Medical University-Tsinghua Medicine College, Taiyuan, Shanxi, China

**Keywords:** deep learning, dual-layer spectral detector CT, lung adenocarcinoma, radiomics, tumor spread through air spaces

## Abstract

**Objective:**

Tumor spread through air spaces (STAS) is associated with increased lung adenocarcinoma recurrence, but it can only be identified postoperatively. Here, a predictive nomogram for detecting preoperative STAS was devised, by combining clinical characteristics with spectral dual-layer detector CT (SDCT)-extracted radiomics (Rad) and deep learning (DL) features.

**Methods:**

A total of 197 surgically resected lung adenocarcinoma patients were divided randomly into training (137) and testing (60) cohorts; clinical data, SDCT images, and tumor tissue samples for histopathological STAS identification were obtained. Rad features were extracted by PyRadiomics, and DL by the ResNet50 convolutional neural network, from manually delineated tumor regions of interest in SDCT, and then incorporated into seven machine learning algorithms; receiver operating characteristic (ROC) analysis identified the best-performing one for the Rad, DL, and DLR (Rad+DL) models. The predictive nomogram was formed by combining DLR with statistically significant clinical characteristics identified by uni- and multivariate logistic regression analyses, and its performance was evaluated by ROC and calibration curve analyses.

**Results:**

Logistic regression was the best-performing machine learning algorithm, and DLR showed relatively better predictive performance than Rad and DL, with areas under the curve (AUCs) of 0.904 for the training and 0.862 for the testing cohort. The nomogram, comprising DLR with the clinical characteristic of pleural indentation, had the highest accuracy, with AUCs of 0.918 for the training and 0.896 for the testing cohort; its predictions strongly corresponded with actual STAS positivity under calibration curve analysis.

**Conclusion:**

The predictive nomogram facilitates reliable preoperative prediction of STAS in lung adenocarcinoma, serving as a valuable tool for devising personalized surgical treatments.

## 1 Introduction

Lung cancer is one of the most malignant tumors, with the most common subtype, at ~55%-60%, being adenocarcinomas (1). The primary treatment for lung adenocarcinoma is surgery (2), but postsurgical recurrence and distant metastases rates remain high, owing to elevated tumor heterogeneity and invasiveness (3). One significant invasive pathological characteristic is tumor spread through air spaces (STAS), which has been included as part of the pathological invasion pattern of lung cancer by the WHO since 2015 (4). Indeed, STAS has been associated with the poor prognoses of lung adenocarcinomas; it was linked to significantly increased risk for local recurrence, which was most pronounced among patients who underwent sub-lobar resection (5, 6). However, applying STAS as a potential marker for preoperative risk assessment remains limited, as its diagnosis relies on postoperative histopathological examination (4). Therefore, developing strategies to preoperatively identify STAS is of great importance, as it could serve as a potential non-invasive predictive tool to detect high-risk patients, subsequently facilitating personalized treatments.

Radiomics (Rad) and deep learning (DL) algorithms are becoming widely used for tumor phenotyping and prognostic assessment. Rad can extract high-dimensional quantitative features from CT images, such as texture, shape, and density, which reflect tumoral internal structure and heterogeneity (7, 8). Likewise, DL algorithms can automatically identify deep features in images, facilitating the “mining” of more discriminative information (9, 10). Based on these capabilities, features obtained from the combination of these two technologies could potentially yield more comprehensive, accurate tumor characterizations. With respect to STAS, studies have developed Rad feature-based predictive models, which have demonstrated a degree of clinical applicability (11, 12); the features, however, were mainly extracted from conventional CT and have not effectively integrated DL features, or clinical characteristics. These limitations could be mitigated with spectral dual-layer detector CT (SDCT), which uses two layers of detectors to synchronously collect high- and low-energy X-ray data. SDCT could generate a variety of energy spectrum-based images, such as virtual single energy, iodine density, effective atomic number, and electron cloud density images, based on conventional CT (13). It also has anti-correlated noise suppression capabilities, reducing noise, along with improving image quality and stability (14). In fact, multiple studies have demonstrated that SDCT has good diagnostic value for differentiating benign versus malignant lung nodules, identifying lung cancer subtypes, and predicting programmed death-ligand 1 expression (15–17). Based on these findings, applying Rad to SDCT images could enable the extraction of additional features that more fully reflect the heterogeneity of the tumor and its surrounding tissues, in turn enhancing their ability to accurately model pathological conditions (18, 19).

In this study, we extracted Rad and DL features from SDCT images, which, along with clinical characteristics, were incorporated into a predictive nomogram for STAS positivity in lung adenocarcinoma. The nomogram showed relatively better predictive performance than clinical, Rad, or DL features alone for preoperatively predicting STAS, thereby providing a more precise predictor.

## 2 Materials and methods

### 2.1 Patient recruitment and obtaining clinical characteristics data

**Figure 1:**
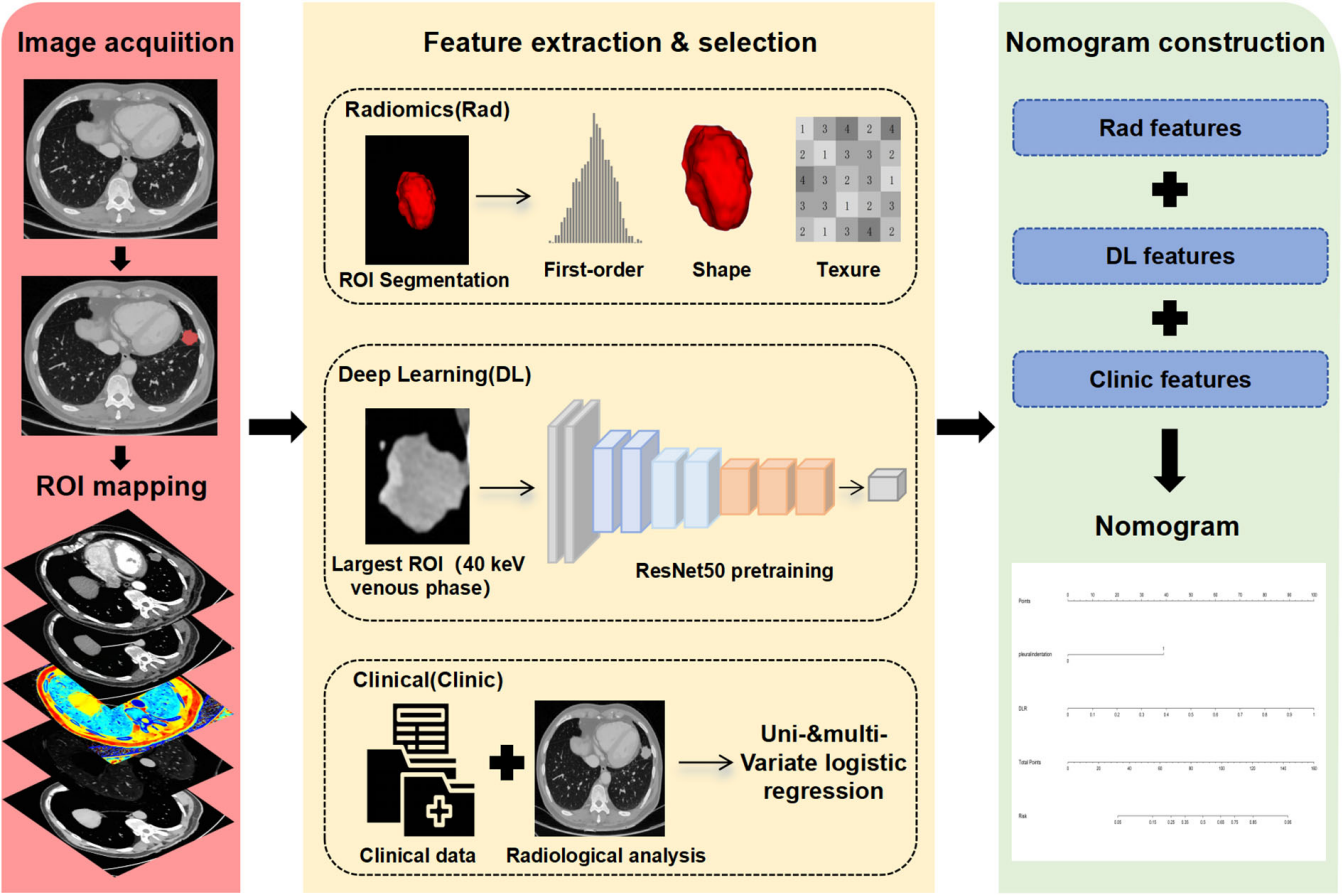
The overall workflow of the study.

**Figure 2:**
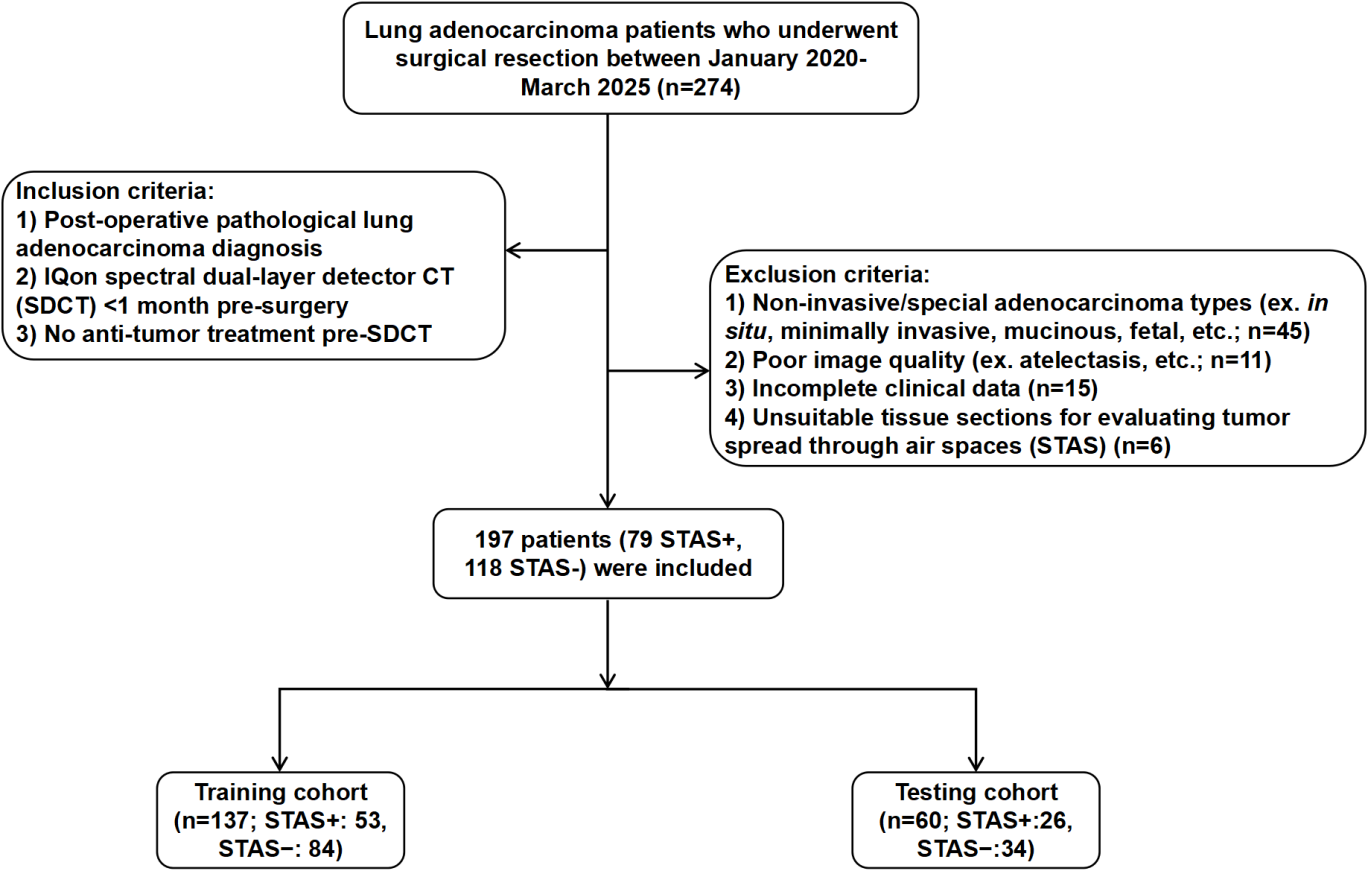
Flowchart showing patient enrollment and division into training and testing cohorts.

Lung adenocarcinoma patients, who underwent surgical resection at the First Hospital of Shanxi Medical University between January 2020-March 2025, were retrospectively analyzed for this study. The study was approved by the Ethics Committee of the First Hospital of Shanxi Medical University. The overall workflow of the study is illustrated in **Figure 1**. **Figure 2** outlines the inclusion and exclusion criteria; after their application, 197 patients were included (79 STAS-positive, 118 STAS-negative) and randomly allocated to the training and validation cohorts at an approximate ratio of 7:3, resulting in 137 patients in the training cohort (53 STAS-positive, 38.7%) and 60 patients in the validation cohort (26 STAS-positive, 43.3%). Clinical characteristics, including age, sex, smoking status, lung cancer family history (LCFH), preoperative carcinoembryonic antigen (CEA), nodule location, and operation type, were obtained from electronic medical records and the picture archiving system.

### 2.2 STAS histopathological assessment

Lung adenocarcinoma specimens were paraffin-embedded and stained with hematoxylin and eosin, and sections were independently assessed for STAS by two experienced pathologists blinded to the SDCT findings. Subtypes were identified based on the 2021 WHO lung cancer histological classification. STAS was defined as tumor cells spreading into alveolar spaces around the main tumor, in the form of micropapillary clusters, solid cancer nests, or single cells separated from the main tumor (4) STAS positivity was defined as STAS presence in any section. Inconsistencies between the two pathologists were resolved by a third senior pathologist.

### 2.3 SDCT image acquisition, analysis, and image segmentation

Virtual plain and enhanced scans were obtained from all patients by IQon Spectral CT (Philips Healthcare). Patients lay in a supine position, and images, covering lung apices to bases, were acquired at the end of a breath-hold. Scanning parameters were as follows: tube voltage 120 kVp, tube current controlled by automatic modulation, rotation time 0.5 s/r, and pitch 0.969. For enhanced scans, iodixanol (iopromide, 1.0-1.2 mL/kg) was injected with a high-pressure syringe, at 2.5-3.5 mL/s, through the median cubital vein, followed by 40-mL normal saline injection at the same flow rate. Arterial and venous phase images were acquired at 25–30 s and 65–70 s, respectively, post-contrast agent injection.

TNM staging was conducted on CT images, based on the American Joint Committee on Cancer staging system (8^th^ ed.). CT features included nodule density (ex. pure ground glass, part-solid, pure solid), tumor size, consolidation tumor ratio (CTR), edge characteristics (ex. spiculated, lobulated), plus the presence of cavitation, pleural indentation, and air bronchogram. CT evaluations were independently performed by two chest radiologists with >10-year experience; inconsistencies between the 2 were resolved by consultation.

For SDCT image segmentation, venous phase (40 keV single-energy) images were imported into ITK-SNAP (3.8.0) (20), and tumor boundaries were manually delineated, layer by layer, by a radiologist with a 5-year experience in thoracic diagnosis and blinded to the clinical and histopathological findings, to obtain a 3D volume of interest (VOI). To ensure that adjacent normal tissues and blood vessels were excluded when outlining the VOI, the radiologist referred to coronal and sagittal images to clarify the spatial relationship around the lesion. The same VOI delineation process was repeated 30 days later for 30 randomly selected patients by the same radiologist to assess intra-observer reproducibility. Radiomics features were re-extracted from the repeated segmentations, and the intraclass correlation coefficient (ICC) was calculated for each feature to evaluate feature stability.

### 2.4 Rad and DL feature extraction

Rad features were extracted from the regions of interest (ROIs) using the PyRadiomics (version 3.0.1) with a predefined parameter configuration file (21), yielding 1,403 features falling into three categories: 1) geometric (n=23), reflecting the 3D morphological properties of the tumor, 2) intensity (n=340), representing the grayscale distribution characteristics of voxels in the tumor area, and 3) texture (n=1,040), including advanced ones, such as gray-level co-occurrence, run-length, size zone, and neighboring grayscale difference matrices, which all characterize the spatial structure pattern in the images (22). During feature extraction, image normalization was applied, images were resampled to an isotropic voxel spacing of 3 × 3 × 3 mm, and gray-level discretization was performed with a bin width of 5.

DL features were extracted by using the largest “slice” of the VOI, representing the ROI; there, the image containing the minimum bounding rectangle of the ROI was cropped and resized to 256×256 pixels by linear interpolation. Data augmentation was performed on the images during the training phase, including random horizontal and vertical flipping, and then cropping to 224×224 pixels to improve the generalization ability of the model. The ResNet50 convolutional neural network (CNN) was pretrained on the ImageNet data. As CT images are single-channel grayscale images, they were converted into three-channel images using a convert2rgb operation before being input into the network. Transfer learning was then applied by fine-tuning the model parameters on the training set. During training, stochastic gradient descent (SGD) was used as the optimizer with a cosine decay learning rate schedule. The maximum learning rate was set to 0.01, the batch size was 32, and the model was trained for 40 epochs. Afterward, DL features were extracted from the global average pooling layer (the penultimate layer), resulting in a 2,048-dimensional feature vector for each image. These features were used for constructing the DLR model.

### 2.5 Establishing the predictive nomogram

The clinical characteristics model (Clinic) was established by subjecting the characteristics to uni- and then multivariate logistic regression analyses to identify independent predictors with statistical significance. Their predictive efficacies were subsequently evaluated by a logistic regression model.

Rad models were devised by standardizing Z-scores for all extracted features and only retaining those with p<0.05 under the Student’s t test. Features were further evaluated by the Pearson correlation coefficient, in which for any two with coefficients >0.9, 1 was removed to reduce collinearity risk. Afterward, least absolute shrinkage and selection operator (LASSO) regression was used for 10-fold cross-validation, to identify the optimal regularization parameter λ, and subsequently, features with the greatest predictive value and explanatory power. These features were incorporated into seven machine learning algorithms: logistic regression (LR), support vector machine (SVM), random forest (RF), gradient boosting tree (XGBoost), supervised learning ensemble (LightGBM), multilayer perceptron neural network (MLP), and K nearest neighbor algorithm (KNN), of which the best-performing was selected for Rad.

For DL, the pretrained ResNet50 convolutional neural network (CNN) was used. Tumor images were input into the network, and predictions were generated directly by the ResNet50 model.

For DLR, early fusion (feature-level fusion) was applied by combining radiomics features with DL features to form a fusion feature set. The DLR model was then built using the same seven machine learning algorithms as those used in the radiomics analysis, and the optimal algorithm was selected.

Clinic, Rad, DL, and DLR predictive capabilities were evaluated by receiver operating characteristic curve (ROC) analyses, whereas the calibration of the model fit was assessed by calibration curves. Models with the greatest predictive capabilities were incorporated into the predictive nomogram for STAS.

### 2.6 Statistical analysis

All analyses were performed by SPSS software (27.0). Continuous variables are presented as mean ± standard deviation, if normally distributed, and comparisons between two groups were performed by the independent sample t test. For non-normally distributed continuous variables, they were presented as median (interquartile range), and comparisons between two groups were performed by the Mann–Whitney U test. Categorical variables were expressed as frequencies (percentages) and assessed by χ² or Fisher’s exact test. P<0.05 was considered statistically significant.

## 3 Results

### 3.1 Analyzing patient clinical characteristics and establishing the clinic model

No significant differences were present between training and testing cohorts for the clinical characteristics of age, sex, smoking status, LCFH, CEA, operation type, TNM stage (**Supplementary Table S1**), plus CT imaging features regarding nodule location and density, largest tumor (LD) and maximum solid diameters, CTR, and presence/absence of spiculation, lobulation, cavitation, air bronchogram, and pleural indentation (**Supplementary Table S1**). However, within the training cohort, significant differences were present between STAS-positive and -negative patients for sex, LCFH, nodule density, LD, maximum solid diameter, CTR, and pleural indentation (**Table 1**), in which STAS positivity was significantly more prevalent among men, without LCFH and pleural indentation, plus having pure solid nodules, along with larger LD, maximum solid diameter, and CTR (**Table 1**). Based on these findings, we conducted univariate logistic regression analysis, where sex, nodule density, maximum solid diameter, CTR, and pleural indentation were significantly associated with STAS positivity (**Table 2**). However, under multivariate logistic regression analysis, pleural indentation was the only significant independent STAS-positivity predictor (**Table 2**). Therefore, pleural indentation was incorporated into the Clinic model, which, under ROC curve analysis, yielded an area under the curve (AUC) of 0.728 (95% confidence interval [CI]=0.665–0.790) in the training and 0.672 (95% CI = 0.581–0.763) in the testing cohort, indicating moderate predictive performance for predicting STAS positivity (**Table 3**).

**Table 1:** Clinical and CT imaging characteristics, between tumor spread through air spaces (STAS)-negative and STAS-positive patients, in the training cohort.

Characteristic	Total (n=137)	STAS-positive (n=53)	STAS-negative (n=84)	*p* value
Age (years)	63.0 (55.0-69.5)	63.0 (56.5-70.0)	63.0 (54.0-69.0)	0.669
Sex (%)				0.041
Female	77 (56.2)	24 (45.3)	53 (63.1)	
Male	60 (43.8)	29 (54.7)	31 (36.9)	
Smoking status (%)				0.751
Never	93 (67.9)	34 (64.1)	59 (70.2)	
Former	18 (13.1)	8 (15.1)	10 (11.9)	
Current	26 (19.0)	11 (20.8)	15 (17.9)	
LCFH (%)				0.021
Absent	4 (2.9)	4 (7.5)	0 (0.0)	
Present	133 (97.1)	49 (92.5)	84 (100.0)	
CEA (µg/L)	4.0 ± 1.7	4.2 ± 2.5	3.9 ± 1.0	0.301
Operation type (%)				0.154
Lobectomy	82 (60.4)	35 (66.0)	47 (56.0)	
Segmentectomy	41 (28.9)	11 (20.8)	30 (35.7)	
Wedge resection	14 (10.7)	7 (13.2)	7 (8.3)	
T stage (%)				0.121
1	121 (88.3)	44 (83.0)	77 (91.7)	
2	15 (11.0)	9 (17.0)	6 (7.1)	
3	1 (0.7)	0 (0.0)	1 (1.2)	
N stage (%)				0.073
0	129 (94.0)	51 (96.2)	78 (92.8)	
1	2 (1.5)	0 (0.0)	2 (2.4)	
2	2 (1.5)	2 (3.8)	0 (0.0)	
3	4 (3.0)	0 (0.0)	4 (4.8)	
TNM stage (%)				0.385
I	113 (82.5)	41 (77.3)	72 (85.7)	
II	3 (2.2)	1 (1.9)	2 (2.4)	
III	20 (14.6)	10 (18.9)	10 (11.9)	
IV	1 (0.7)	1 (1.9)	0 (0.0)	
Nodule location (lobe, %)				0.757
Left upper	31 (22.6)	14 (26.4)	17 (20.2)	
Left lower	23 (16.8)	10 (18.9)	13 (15.5)	
Right upper	41 (29.9)	14 (26.4)	27 (32.1)	
Right middle	6 (4.4)	3 (5.7)	3 (3.6)	
Right lower	36 (26.3)	12 (22.6)	24 (28.6)	
Nodule density (%)				<0.001
Pure ground glass	20 (14.6)	4 (7.6)	16 (19.0)	
Part-solid	59 (43.1)	12 (22.6)	47 (56.0)	
Pure solid	58 (42.3)	37 (69.8)	21 (25.0)	
LD (cm)	1.8 (1.5-2.4)	2.1 (1.6-2.9)	1.7 (1.4-2.2)	0.011
Maximum solid diameter (cm)	1.1 (0.6-1.8)	1.7 (1.1-2.7)	0.8 (0.5-1.3)	<0.001
CTR	0.6 ± 0.4	0.8 ± 0.3	0.5 ± 0.4	<0.001
Spiculation (%)				0.101
Absent	125 (91.2)	51 (96.2)	74 (88.1)	
Present	12 (8.8)	2 (3.8)	10 (11.9)	
Lobulation (%)				0.283
Absent	134 (97.8)	53 (100.0)	81 (96.4)	
Present	3 (2.2)	0 (0.0)	3 (3.6)	
Cavitation (%)				0.400
Absent	31 (22.6)	14 (26.4)	17 (20.2)	
Present	106 (77.4)	39 (73.6)	67 (79.8)	
Air bronchogram (%)				0.283
Absent	3 (2.2)	0 (0.0)	3 (3.6)	
Present	134 (97.8)	53 (100.0)	81 (96.4)	
Pleural indentation (%)				<0.001
Present	91 (66.4)	50 (94.3)	41 (48.8)	
Absent	46 (33.6)	3 (5.7)	43 (51.2)	

Continuous variables are presented as mean ± standard deviation, if normally distributed, or median (interquartile range), if not normally distributed.

LCFH, lung cancer family history; CEA, carcinoembryonic antigen; LD, largest tumor diameter; CTR, consolidation tumor ratio.

**Table 2:** Uni- and multivariate logistic regression analyses of clinical and CT features for tumor spread through air spaces (STAS) positivity in the training cohort.

Characteristic	Univariate analysis		Multivariate analysis	
	OR (95% CI)	*p* value	OR (95% CI)	*p* value
Age (years)	1.016 (0.988-1.045)	0.270		
Sex	2.066 (1.027-4.157)	0.042	2.174 (0.930-5.079)	0.073
Smoking status	1.170 (0.761-1.799)	0.473		
LCFH	——	0.999		
CEA	1.112 (0.904-1.369)	0.314		
Operation type	0.891 (0.532-1.491)	0.660		
T stage	1.799 (0.684-4.734)	0.234		
N stage	0.719 (0.348-1.483)	0.371		
TNM stage	1.394 (0.887-2.191)	0.150		
Nodule location	0.888 (0.701-1.124)	0.324		
Nodule density	3.895 (2.099-7.228)	<0.001	0.990 (0.131-7.496)	0.992
LD (cm)	1.435 (0.986-2.090)	0.060		
Maximum solid diameter (cm)	1.945 (1.369-2.763)	<0.001	1.060 (0.645-1.741)	0.817
CTR	1.024 (1.013-1.036)	<0.001	1.015 (0.978-1.054)	0.435
Spiculation	3.446 (0.724-16.391)	0.120		
Lobulation	——	0.999		
Cavitation/vacuole	1.415 (0.629-3.180)	0.401		
Air bronchogram	——	0.999		
Pleural indentation	17.480 (5.053-60.470)	<0.001	11.377 (3.053-42.403)	<0.001

“——” indicates that the OR could not be estimated between STAS-positive and -negative groups for the trait, as STAS positivity was not present in one of the groups.

OR, odds ratio; CI, confidence interval; LCFH, lung cancer family history; CEA, carcinoembryonic antigen; LD, largest tumor diameter; CTR, consolidation tumor ratio.

**Table 3:** Receiver operating characteristic curve results for the predictive accuracy of different models between training and testing cohorts.

Model	AUC (95% CI)	Accuracy	Sensitivity	Specificity	PPV	NPV
Training						
Clinic	0.728 (0.665-0.790)	0.679	0.943	0.512	0.549	0.935
Rad	0.868 (0.806-0.931)	0.810	0.774	0.833	0.745	0.854
DL	0.880 (0.819-0.942)	0.839	0.830	0.845	0.772	0.887
DLR	0.904 (0.855-0.953)	0.847	0.811	0.869	0.796	0.880
Nomogram	0.918 (0.871-0.965)	0.876	0.830	0.905	0.846	0.894
Testing						
Clinic	0.672 (0.581-0.763)	0.633	0.962	0.382	0.543	0.929
Rad	0.838 (0.723-0.953)	0.800	0.769	0.824	0.770	0.823
DL	0.852 (0.747-0.956)	0.800	0.846	0.765	0.734	0.867
DLR	0.862 (0.769-0.956)	0.800	0.808	0.794	0.750	0.844
Nomogram	0.896 (0.817-0.975)	0.833	0.846	0.824	0.786	0.875

Clinic, clinical model; Rad, radiomics model; DL, deep learning model; DLR, deep learning-radiomics model; AUC, area under the curve; CI, confidence interval; PPV, positive predictive value; NPV, negative predictive value.

### 3.2 LR yielded the most optimal Rad and DLR models for STAS positivity

Out of the 1,403 radiomics features extracted, the ICC analysis showed good stability. There were 16 incorporated into the Rad model, after applying Z-score normalization, t test, Spearman correlation analysis to remove redundancies (threshold r>0.9), and LASSO regression. **Supplementary Figure S1** shows the LASSO results, including the regression path diagram (**Supplementary Figure S1A**), 10-fold cross validation to identify the optimal λ value (**Supplementary Figure S1B**), and the weights of the 16 incorporated features (**Supplementary Figure S1C**).

**Figure 3:**
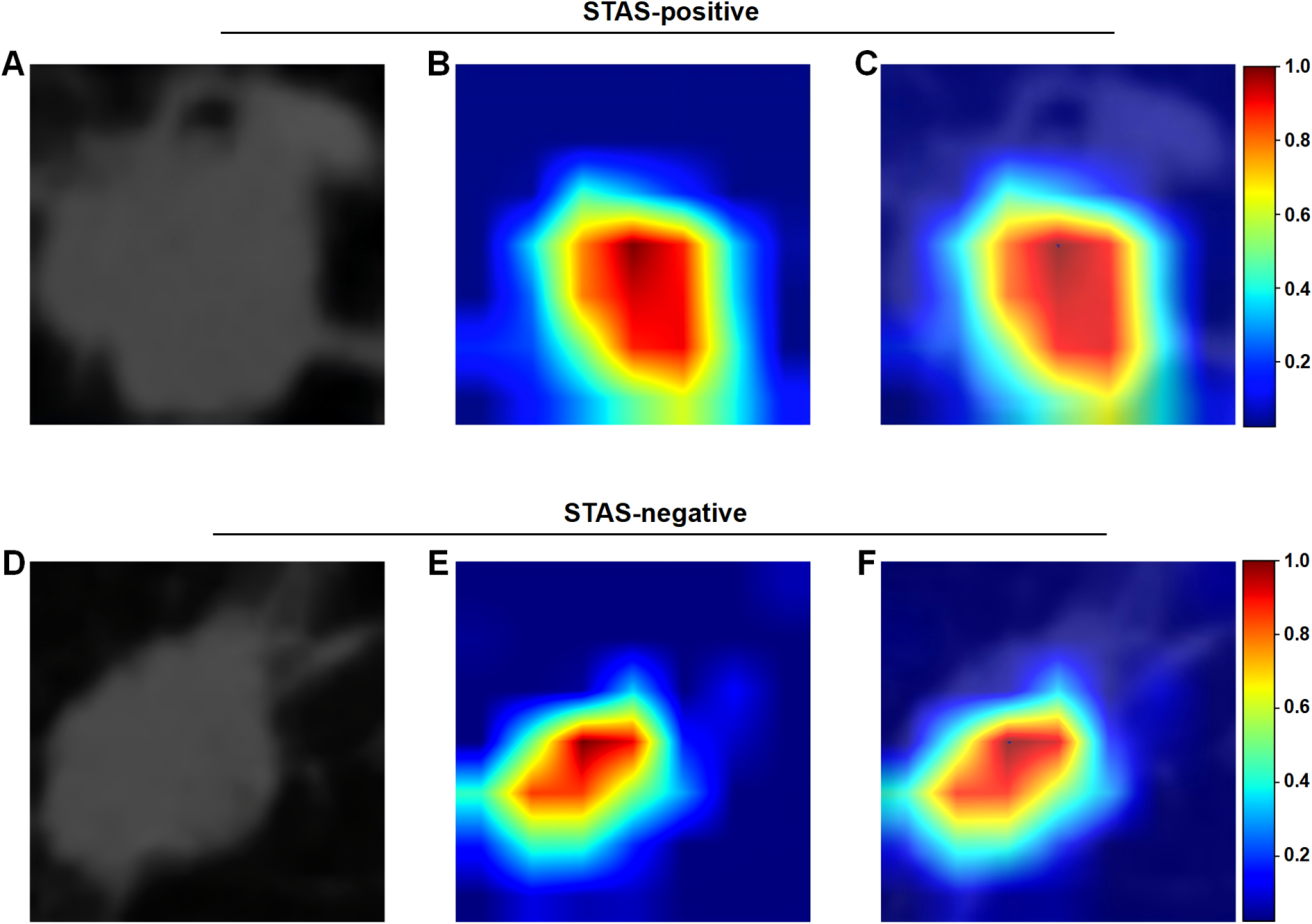
Gradient-weighted class activation mapping for tumor spread through air spaces (STAS)-positive and -negative lung adenocarcinoma patients, based on 40-keV single-energy venous phase images from spectral dual-layer detector CT (SDCT). **(A)** Original SDCT image with tumor region of interest (ROI), **(B)** heatmap, and **(C)** overlay of the heatmap on the SDCT image for a STAS-positive patient. **(D)** Original SDCT image with tumor ROI, **(E)** heatmap, and **(F)** overlay of the heatmap on the SDCT image for a STAS-negative patient. Red areas represent focus areas for high-order deep learning (DL) features within the tumor ROI.

Similarly, the ResNet50 CNN extracted 2,048 DL features, which were subjected to dimensionality reduction using principal component analysis and then fused with radiomics features, via LASSO regression analysis, to yield 14 features for the DLR model (**Supplementary Figures S2A–C**). The accuracy of DL features for predicting STAS positivity was further confirmed by gradient-weighted class activation mapping, which visualized the areas of focus for these high-order features within the ROI; these areas, as shown in **Figure 3**, were mainly found in solid, as well as complex edge morphological areas of the tumor, suggesting that these areas may serve as the key locale for identifying predictive features in SDCT to distinguish between STAS positivity (**Figures 3A–C**) versus -negativity (**Figures 3D–F**).

**Figure 4:**
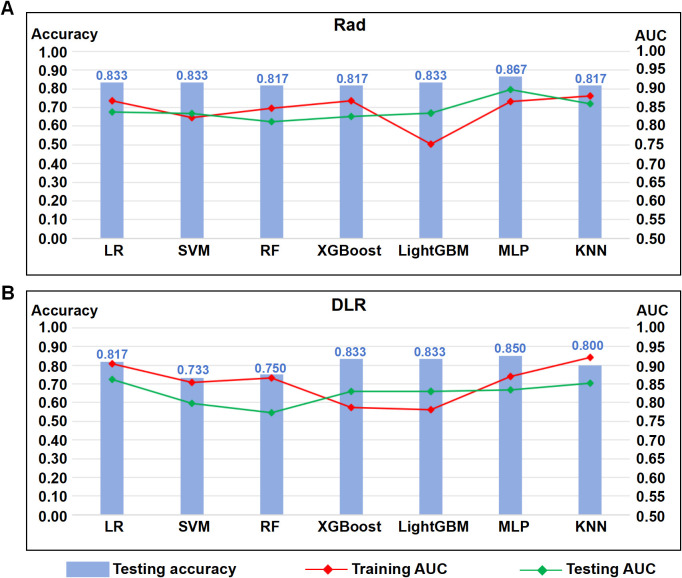
Comparisons between seven machine learning algorithms incorporating radiomics (Rad), or a combination of Rad and DL (DLR) features, for the training and testing cohorts. **(A)** Combination chart showing areas under the curve (AUCs) for logistic regression (LR), support vector machine (SVM), random forest (RF), gradient boosted tree (XGBoost), supervised learning ensemble (LightGBM), multilayer perceptron neural network (MLP), and K nearest neighbor (KNN) machine learning algorithms, incorporating Rad features, between the training (red) and testing (green) cohorts, plus predictive accuracies for the testing cohort (blue). **(B)** Combination chart showing AUCs for the seven machine learning algorithms, incorporating DLR features, between the training and testing cohorts, plus predictive accuracies for the testing cohort.

Both Rad and DLR features were incorporated into the seven machine learning algorithms, and their accuracies, for both training and testing cohorts, were assessed by ROC curve analyses. A high degree of accuracy for predicting STAS positivity was present for both sets of seven algorithms, with Rad or DLR features, for the training cohort. However, for the testing cohort, there is a risk of overfitting, owing to the larger gap present between testing and training AUCs for some of the algorithms, such as LightGBM with Rad features (**Figure 4A**), or RF for DLR features (**Figure 4B**). Nevertheless, the most optimal algorithm, with the smallest gap between training and testing cohort AUCs, for both Rad and DLR features, was LR (**Figures 4A, B**), which was thereby selected for constructing Rad, DL, and DLR predictive models.

### 3.3 Nomogram combining clinic and DLR showed the best predictive performance for predicting STAS

**Figure 5:**
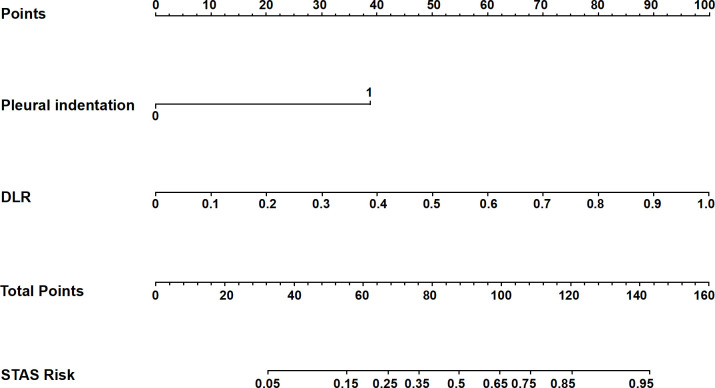
Predictive nomogram for STAS-positivity risk, combining the clinical characteristic of pleural indentation and the DLR model, itself entailing DLR features being incorporated into the LR machine-learning algorithm.

**Figure 6:**
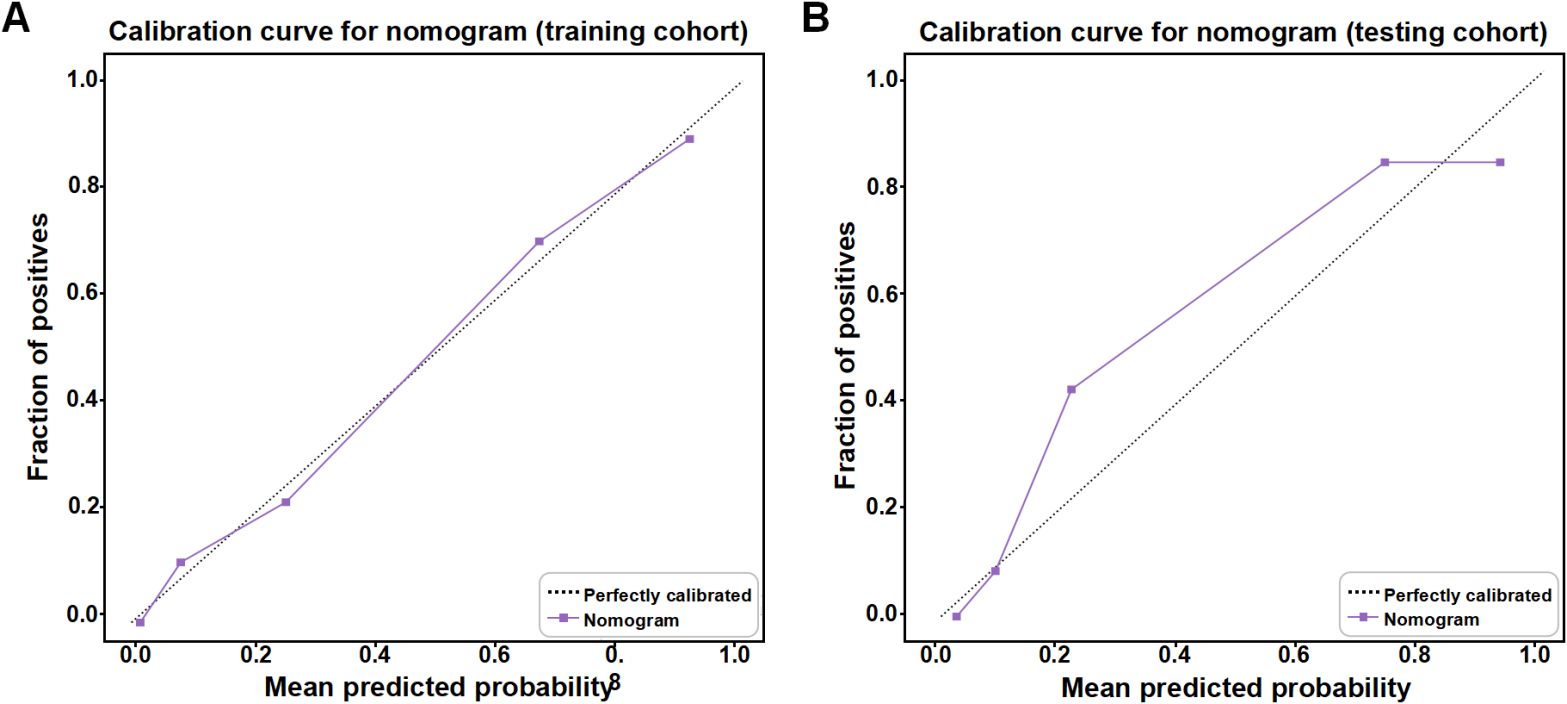
Calibration curves for the predictive nomogram to assess the correspondence between its predictions and actual STAS-positivity occurrence, for **(A)** training and **(B)** testing cohorts.

The predictive nomogram was formulated by combining the Clinic model, containing pleural indentation, with the DLR model incorporating the 14 DLR features into the LR algorithm (**Figure 5**). Pleural indentation presence/absence and DLR scores corresponded to specific point values, whose sum was associated with differing STAS-positivity likelihoods. It is worth noting that the DLR model was selected owing to the fact that it showed higher AUC values in ROC analysis than for either Rad or DL, at 0.904 (95% CI = 0.855–0.953) for the training and 0.862 (95% CI = 0.769–0.956) for the testing cohort (**Table 3**). Furthermore, the nomogram showed the highest predictive performance for predicting STAS positivity, with AUCs of 0.918 (95% CI = 0.871–0.965) for the training and 0.896 (95% CI = 0.817–0.975) for the testing cohort (**Table 3**), higher than for all other predictive models. In addition, we used the Delong test to compare the AUC of each model. The results are provided in **Supplementary Figure S3**. The results showed that the Rad, DL, DLR, and Nomogram models were significantly better than the clinical models (p<0.05) in the training set and validation set, suggesting that the model based on image features had better discriminative ability. However, in the pairwise comparisons between the single models (Rad, DL, and DLR), the differences were not statistically significant, indicating that different modeling strategies had similar performance in predicting STAS. Calibration curve analyses found that a high degree of correspondence was present between predicted and actual occurrences of STAS positivity for the predictive nomogram in the training cohort. In the testing cohort (**Figure 6B**), the calibration curve also demonstrated an overall acceptable agreement with the ideal reference line. In addition, the Hosmer–Lemeshow goodness-of-fit test indicated no significant lack of fit (P > 0.05). Therefore, the predictive nomogram could serve as a useful tool for preoperatively identifying STAS-positive lung adenocarcinoma, in turn aiding the development of effective surgical strategies.

## 4 Discussion

STAS has a fair degree of prevalence in lung cancer, occurring in ~22.9% of stage I, leading to it being considered an independent prognostic factor for poor recurrence-free and overall survival (23, 24). In fact, studies have noted that STAS-positive patients had significantly higher recurrence risk post-sub-lobar resection, leading to them being more suited for lobectomy to lower this risk (25, 26). Consequently, preoperatively identifying STAS occurrence could aid in developing tailored treatment approaches. In this study, we developed such a strategy in the form of a predictive nomogram, which incorporates the clinical characteristic of pleural indentation with 14 DLR features, comprising a combination of features extracted from SDCT images via radiomics and ResNet50, which improved the predictive performance for STAS positivity than Clinic, Rad, DL, and DLR models alone. Therefore, this nomogram could serve as a non-invasive method for preoperative STAS screening in lung adenocarcinomas.

Numerous studies have identified associations between multiple CT features and STAS presence, such as Toyokawa et al., who identified a positive correlation between tumor diameter and STAS positivity (27), and Yin et al., who demonstrated that nodule density has potential in predicting STAS (28). Furthermore, lung cancer-associated features, including spiculation, pleural indentation, and air bronchogram, have been linked to STAS-positivity (27, 29). Our findings agreed with these studies, as we also found significant differences between STAS-positive and -negative patients for pleural indentation under multivariate analysis; this may be due to it reflecting tumor adhesion and local infiltration into the pleura, which could exert a “pulling” effect on surrounding tissues (30).

Owing to SDCT being able to provide multimodal images, including iodine density, effective nuclear charge, and electron cloud density, it has become an emergent quantitative method for analyzing tumor structures and compositions (31). In fact, in this study, 40-keV venous phase single-energy images were selected for Rad and DL feature extraction. This is due to lower-energy images possessing higher attenuation coefficients for iodine contrast agents, subsequently significantly improving lesion contrast and enabling clear tumor boundary delineation (32, 33). Moreover, compared with arterial, venous phase images more stably reflect blood supply statuses and microcirculatory perfusion of tumor parenchyma and peritumoral tissues, leading to higher signal-to-noise ratios and morphological stability, thus rendering them more suitable for feature extraction (34, 35).

Rad has been widely used to assess malignant tumor degree of differentiation, in turn enabling preoperative risk stratification (7), and some clinicians have applied it for predicting STAS in lung adenocarcinoma. For instance, Jiang et al. extracted Rad features from conventional CT and incorporated them into an RF-based predictive algorithm, yielding a moderately high AUC of 0.754 (11). The LR-based Rad model in this study, incorporating SDCT-extracted features, yielded improved predictive results, with AUCs of 0.868 and 0.838 for the training and testing cohorts, respectively. Along with Rad features, other studies, such as Han et al., have also demonstrated that adding high-dimensional features, extracted by DL algorithms, could mitigate conventional Rad shortcomings in spatial pattern recognition, thereby improving predictive model accuracy and stability (12). Indeed, their model, combining Rad and clinical features, had an AUC of 0.865, which was fairly close to the AUCs of 0.918 and 0.896, for the training and testing cohorts, respectively, achieved by the predictive nomogram in this study, which incorporated clinical characteristics and DLR features. Furthermore, the nomogram model achieved the highest AUC in both cohorts, but its performance improvement was not statistically significant compared with Rad or DL. This result may be due to the dominance of Rad features in the nomogram, which already have strong discriminative ability, thus weakening the gain of multimodal fusion to a certain extent. It is worth noting that for DL, a 2D CNN method, ResNet50, was selected for this study, in order to avoid overfitting problems associated with complex 3D CNN when trained on small sample sizes (36). In our preliminary exploration, we attempted to construct models based on 3D CNNs; however, under the limited sample size, they exhibited marked overfitting and instability. In contrast, the simpler structure and higher computational efficiency of ResNet50 improved model stability, along with maintaining good interpretability and practicality.

In summary, we successfully constructed a predictive nomogram for STAS positivity in lung adenocarcinoma, by combining the clinical characteristic of pleural indentation with SDCT-extracted Rad and DL features. These features were incorporated into seven machine learning algorithms, and LR was found to be the most optimal for the resulting Rad, DL, and DLR models. The nomogram showed relatively better predictive performance than the Clinic, Rad, DL, and DLR models for preoperatively predicting STAS positivity, providing an effective, non-invasive STAS detection method in lung cancer patients, and facilitating personalized surgical strategy development. However, several limitations should be acknowledged. Firstly, this was a single-center retrospective study with a relatively limited sample size, which may introduce unavoidable selection bias. Future multicenter, prospective studies are warranted to further validate the generalizability of the proposed model. Secondly, due to the sample size, we adopted a 2D maximum-slice approach for deep learning feature extraction. Although this method has been widely used in thoracic imaging research and can capture representative tumor features, it may not fully reflect the three-dimensional structure and spatial heterogeneity of tumors. Future studies with larger datasets may explore 3D-CNN architectures to improve spatial representation and predictive performance. In addition, ROI/VOI delineation in this study was performed manually, which may be subject to inter-observer variability. Semiautomatic or automatic segmentation methods should be considered in future work to improve feature stability and reproducibility. Furthermore, the study population was limited to lung adenocarcinoma, without including other pathological types of lung cancer, and subgroup analyses according to different clinical stages were not performed. Future studies may further integrate relevant molecular characteristics and serological biomarkers and conduct stratified analyses across different pathological types and clinical stages to further refine the model and improve its clinical applicability.

## Data Availability

The original contributions presented in the study are included in the article/**Supplementary Material**. Further inquiries can be directed to the corresponding authors.
