# Supplementary material for: Nomogram combining spectral dual-layer detector CT radiomics and deep learning features predicts alveolar tumor spread
Source: Front Oncol. 2026 Apr 10;16:1703790. doi: 10.3389/fonc.2026.1703790 (PMC13106009; doi:10.3389/fonc.2026.1703790)
Supplement: Supplementary file 1 [file DataSheet1.docx]

**Supplementary Materials**

for

**Nomogram combining spectral dual-layer detector CT radiomics and deep learning features predicts alveolar tumor spread**

**Supplementary Table**

**Table S1** Clinical and CT features between training and testing cohorts

| Feature | Total (n=197) | Training cohort (n=137) | Testing cohort  (n=60) | p value |
| --- | --- | --- | --- | --- |
| Age (years) | 63.0 (55.0-69.0) | 63.0 (55.0-69.5) | 60.0 (55.0-68.0) | 0.371 |
| Sex (%) |  |  |  | 0.217 |
| Female | 105 (53.3) | 77 (56.2) | 28 (46.7) |  |
| Male | 92 (46.7) | 60 (43.8) | 32 (53.3) |  |
| Smoking status (%) |  |  |  | 0.627 |
| Never | 136 (69.0) | 93 (67.9) | 43 (71.7) |  |
| Former | 23 (11.7) | 18 (13.1) | 5 (8.3) |  |
| Current | 38 (19.3) | 26 (19.0) | 12 (20.0) |  |
| LCFH (%) |  |  |  | 0.437 |
| Absent | 7 (3.6) | 4 (2.9) | 3 (5.0) |  |
| Present | 190 (96.4) | 133 (97.1) | 57 (95.0) |  |
| CEA (µg/L) | 4.3±3.0 | 4.0±1.7 | 4.8±4.8 | 0.083 |
| Operation type (%) |  |  |  | 0.879 |
| Lobectomy | 119 (60.4) | 82 (59.9) | 37 (61.6) |  |
| Segmentectomy | 57 (28.9) | 41 (29.9) | 16 (26.7) |  |
| Wedge resection | 21 (10.7) | 14 (10.2) | 7 (11.7) |  |
| T stage (%) |  |  |  | 0.355 |
| 1 | 172 (87.3) | 121 (88.3) | 51 (85.0) |  |
| 2 | 22 (11.2) | 15 (11.0) | 7 (11.7) |  |
| 3 | 3 (1.5) | 1 (0.7) | 2 (3.3) |  |
| N stage (%) |  |  |  | 0.216 |
| 0 | 183 (92.9) | 129 (94.0) | 54 (90) |  |
| 1 | 3 (1.5) | 2 (1.5) | 1 (1.7) |  |
| 2 | 6 (3.1) | 2 (1.5) | 4 (6.6) |  |
| 3 | 5 (2.5) | 4 (3.0) | 1 (1.7) |  |
| TNM stage (%) |  |  |  | 0.202 |
| I | 159 (80.7) | 113 (82.5) | 46 (76.7) |  |
| II | 8 (4.1) | 3 (2.2) | 5 (8.3) |  |
| III | 29 (14.7) | 20 (14.6) | 9 (15.0) |  |
| IV | 1 (0.5) | 1 (0.7) | 0 (0.0) |  |
| Nodule location (lobe, %) |  |  |  | 0.074 |
| Left upper | 53 (26.9) | 31 (22.6) | 22 (36.7) |  |
| Left lower | 32 (16.2) | 23 (16.8) | 9 (15.0) |  |
| Right upper | 61 (31.0) | 41 (29.9) | 20 (33.3) |  |
| Right middle | 9 (4.6) | 6 (4.4) | 3 (5.0) |  |
| Right lower | 42 (21.3) | 36 (26.3) | 6 (10.0) |  |
| Nodule density (%) |  |  |  | 0.335 |
| Pure ground glass | 26 (13.2) | 20 (14.6) | 6 (10.0) |  |
| Part-solid | 81 (41.1) | 59 (43.1) | 22 (36.7) |  |
| Pure solid | 90 (45.7) | 58 (42.3) | 32 (53.3) |  |
| LD (cm) | 1.8 (1.4-2.5) | 1.8 (1.5-2.4) | 1.9 (1.4-2.9) | 0.979 |
| Maximum solid diameter (cm) | 1.1 (0.6-2.2) | 1.1 (0.6-1.8) | 1.2 (0.6-2.7) | 0.162 |
| CTR | 0.7±0.4 | 0.7±0.4 | 0.6±0.4 | 0.107 |
| Spiculation (%) |  |  |  | 0.621 |
| Absent | 181 (91.9) | 125 (91.2) | 56 (93.3) |  |
| Present | 16 (8.1) | 12 (8.8) | 4 (6.7) |  |
| Lobulation (%) |  |  |  | 0.999 |
| Absent | 193 (98.0) | 134 (97.8) | 59 (98.3) |  |
| Present | 4 (2.0) | 3 (2.2) | 1 (1.7) |  |
| Cavitation (%) |  |  |  | 0.343 |
| Absent | 41 (20.8) | 31 (22.6) | 10 (16.7) |  |
| Present | 156 (79.2) | 106 (77.4) | 50 (83.3) |  |
| Air bronchogram (%) |  |  |  | 0.999 |
| Absent | 4 (2.0) | 3 (2.2) | 1 (1.7) |  |
| Present | 193 (98.0) | 134 (97.8) | 59 (98.3) |  |
| Pleural indentation (%) |  |  |  | 0.151 |
| Present | 137 (69.5) | 91 (66.4) | 46 (76.7) |  |
| Absent | 60 (30.5) | 46 (33.6) | 14 (23.3) |  |

Continuous variables are presented as mean ± standard deviation, if normally distributed, or median (interquartile range), if not normally distributed.

*LCFH* lung cancer family history, *CEA* carcinoembryonic antigen, *LD* largest tumor diameter, *CTR* consolidation tumor ratio.

**Supplementary Figures**

**
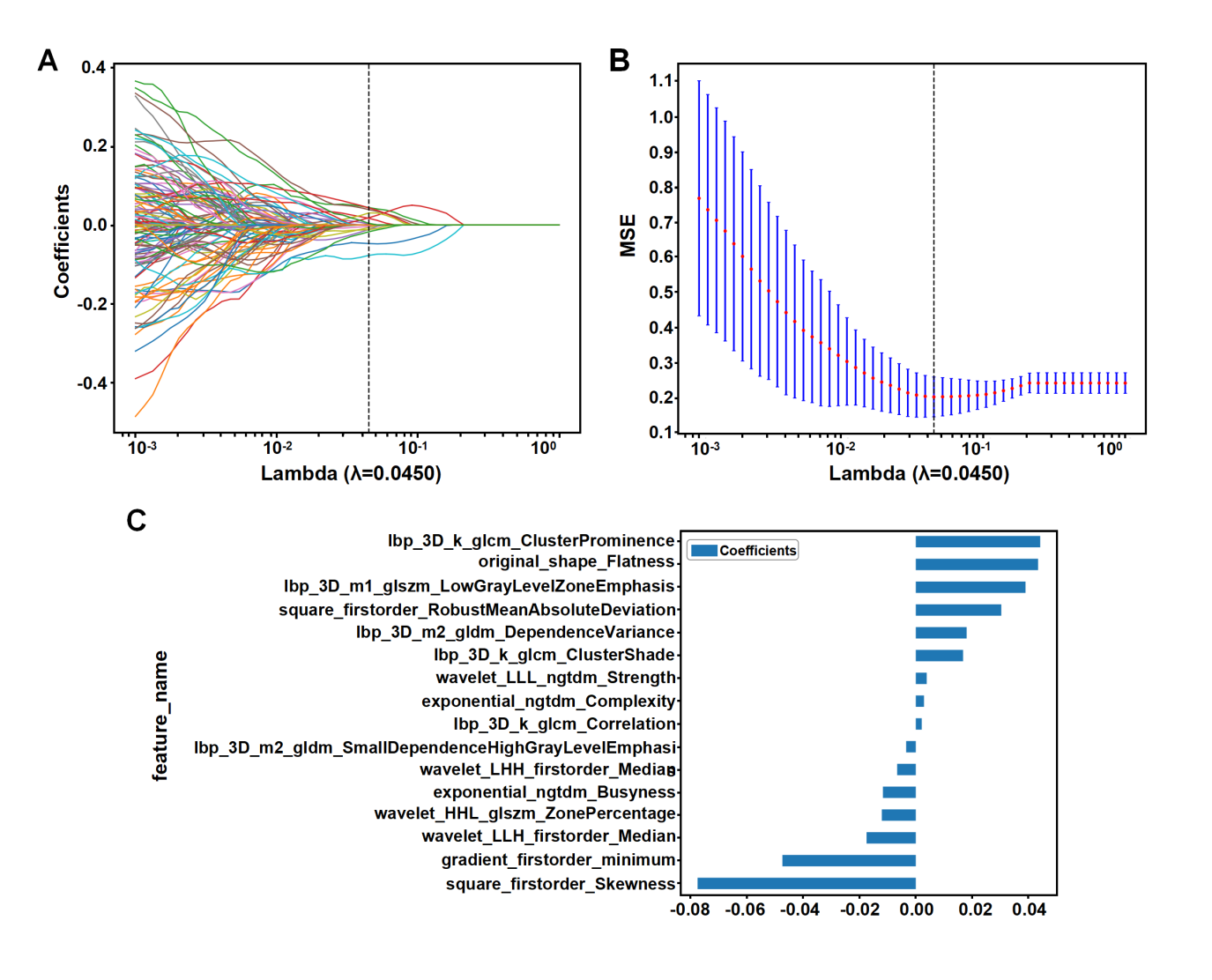
**

**Fig. S1** Least absolute shrinkage and selection operator (LASSO) regression of radiomics (Rad) features for the Rad model. **A** Coefficient path diagram for each of the Rad features, as a function of the regularization parameter log(λ). **B** Line plot showing 10-fold cross validation and minimum mean squared errors (MSE) to determine the optimal λ for LASSO, which was 0.0450. **C** 16 Rad features incorporated into the Rad model, selected based on their (non-zero) regression coefficients, with respect to the optimal λ value.

**
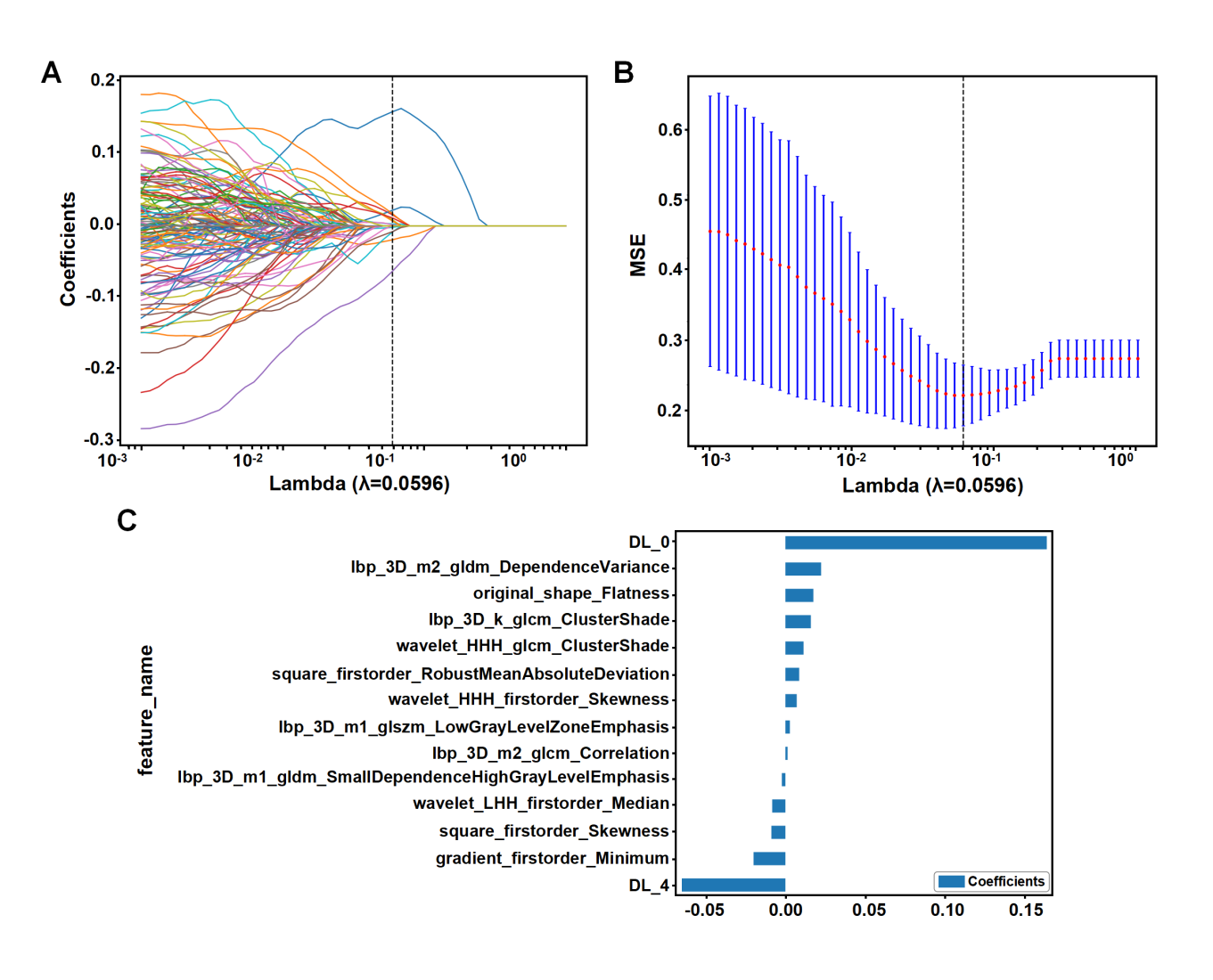
**

**Fig. S2** LASSO regression of deep learning (DL) and Rad features for the deep learning-radiomics (DLR) model. **A** Coefficient path diagram for each of the DLR features, as a function of the regularization parameter log(λ). **B** Line plot showing 10-fold cross validation and MSE to determine the optimal λ for LASSO, which was 0.0596 **C** 14 Rad features incorporated into the Rad model, selected based on their (non-zero) regression coefficients, with respect to the optimal λ value.

**
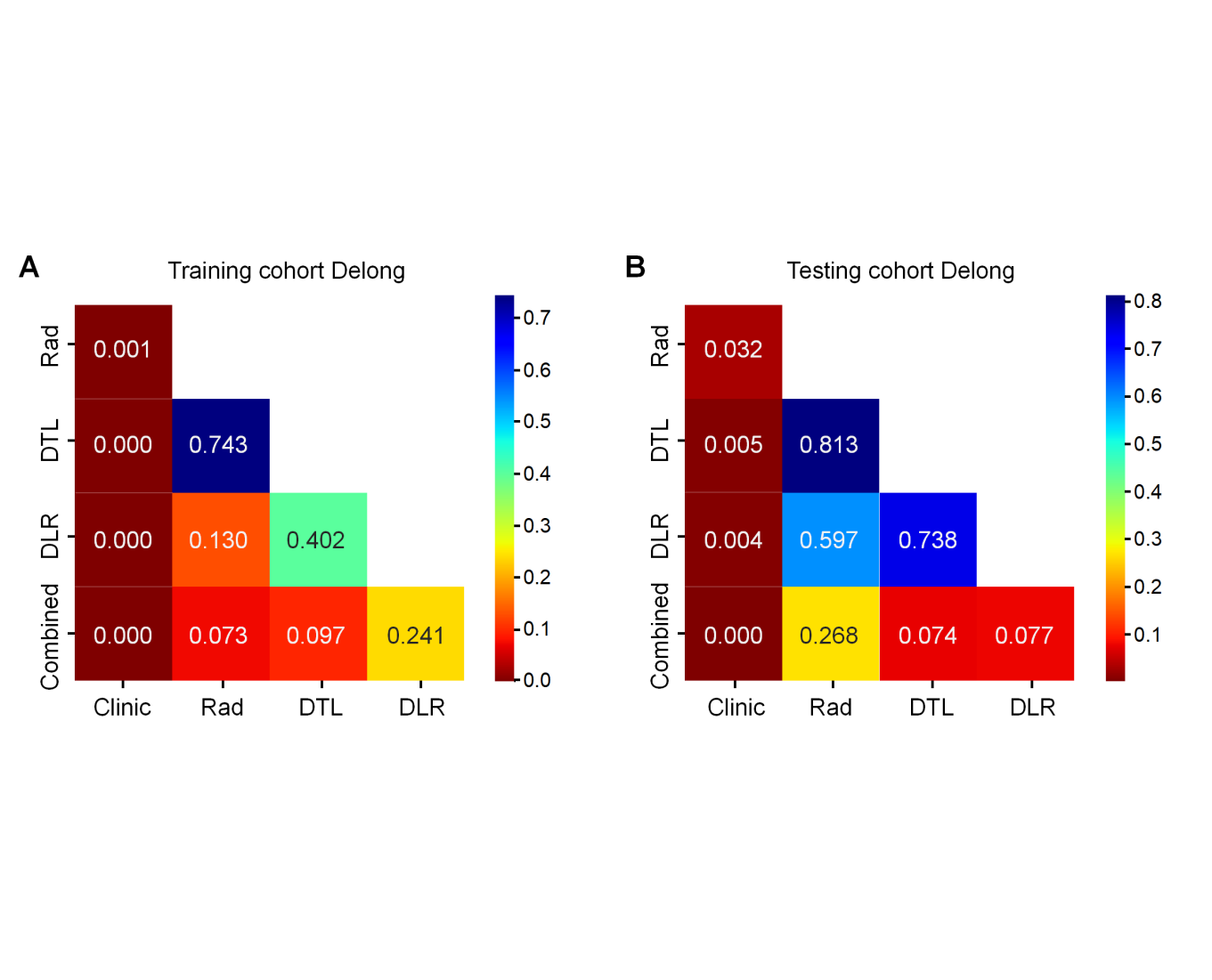
**

**Fig. S3** Pairwise DeLong test results for AUC comparisons among different models. A Training cohort, B Testing cohort. Numbers in the heatmap represent the P values of pairwise comparisons, with color shading indicating the degree of statistical significance.
